# Research on DOA Estimation Based on Acoustic Energy Flux Detection Using a Single MEMS Vector Hydrophone

**DOI:** 10.3390/mi12020168

**Published:** 2021-02-08

**Authors:** Xiaoyong Zhang, Guojun Zhang, Zhenzhen Shang, Shan Zhu, Peng Chen, Renxin Wang, Wendong Zhang

**Affiliations:** 1 State Key Laboratory of Dynamic Testing Technology, North University of China, Taiyuan 030051, China; b1706008@st.nuc.edu.cn (Z.S.); b1906065@st.nuc.edu.cn (S.Z.); s1906084@st.nuc.edu.cn (P.C.); wangrenxin@nuc.edu.cn (R.W.); wdzhang@nuc.edu.cn (W.Z.); 2 Department of Intelligence and Automation, Taiyuan University, Taiyuan 030032, China

**Keywords:** MEMS, vector hydrophone, acoustic energy flux, DOA, maximum likelihood estimation

## Abstract

The principle of acoustic energy flux detection method using a single micro electromechanical system (MEMS) vector hydrophone is analyzed in this paper. The probability distribution of acoustic energy flux and the weighted histogram algorithm are discussed. Then, an improved algorithm is proposed. Based on the algorithm, the distribution range of the energy is obtained by a sliding window, the energy center of gravity in the range is considered as the result of direction of arrival (DOA) estimation, and it is proved to be the maximum likelihood estimation of the target direction. The simulation results show that, with the signal to noise ratio (SNR) from −10 dB to 10 dB, the root mean square error (RMSE) of the improved algorithm is reduced by 47.8% on average, and is more accurate in the presence of interference. The experimental results of lake test are consistent with the theory analysis and simulation results.

## 1. Introduction

The hydrophone array is generally adopted in underwater detection for direction of arrival (DOA) estimation, which requires great physical space [1]. A small-sized micro electromechanical system (MEMS) vector hydrophone with low power consumption has been manufactured by North University of China, providing a new access for the development and application of vector hydrophone [2]. The vector hydrophone can measure acoustic pressure and acoustic vibration velocity simultaneously [3]. A single MEMS vector hydrophone is capable of DOA estimation [4], suitable for limited deployment space of equipment. In underwater systems such as aerial buoy, submersible buoy, and unmanned underwater vehicle, the application of detection unit composed of a single MEMS vector hydrophones can make the structure of the system simpler. Hence, the study on DOA estimation algorithm using a single MEMS vector hydrophone is of strongly practical significance.

The average sound intensity algorithm is a common method for DOA estimation for vector hydrophone. It can suppress isotropic incoherent interference and has a small computation burden, but cannot distinguish more than one target [5]. Nehorai proposes an algorithm which can obtain acoustic energy flux in frequency domain, analyzed its performance and the Cramer-Rao bound [6]. The algorithm can distinguish multiple targets by spectrum [7]. Based on the acoustic energy flux in frequency domain, Zhixiang Yao et al. propose a weighted histogram algorithm [8], which accumulates the acoustic energy flux in the same direction to obtain a weighted histogram of the acoustic energy flux. In the algorithm, the direction of peak energy is considered as the DOA estimation result. In addition, there are other algorithms based on power spectrum estimation or subspace analysis, including beam-forming [9], multiple signal classification (MUSIC) [10] and estimation of signal parameters via rotational invariance technique (ESPRIT) [11]. These algorithms have good resolution, but are generally required to solve eigenvalues or eigenvectors, etc., which is a large computation burden. When signal is required to be processed in real time, the requirements for hardware are higher than the weighted histogram algorithm [12].

This paper analyzed the principle of acoustic energy flux detection method using a single MEMS vector hydrophone. Weighted histogram algorithm principle is discussed, and an improved algorithm is proposed. The result of the algorithm is proved to be the maximum likelihood estimation of the target direction. Besides, the simulation and experiment on the performance of algorithm is conducted in this paper. The simulation results show that, with the different signal to noise ratio (SNR) and interference, the improved algorithm has a better performance than original one. The experimental results of lake test are consistent with theory analysis and the simulation results. All the discussions in this paper assume that the distance between the hydrophone and the sound source is enough to satisfy the far-field condition and the noise in the environment is isotropic white Gaussian noise.

## 2. MEMS Vector Hydrophone and Acoustic Energy Flux Detection

### 2.1. The Design of MEMS Vector Hydrophone

**Figure 1:**
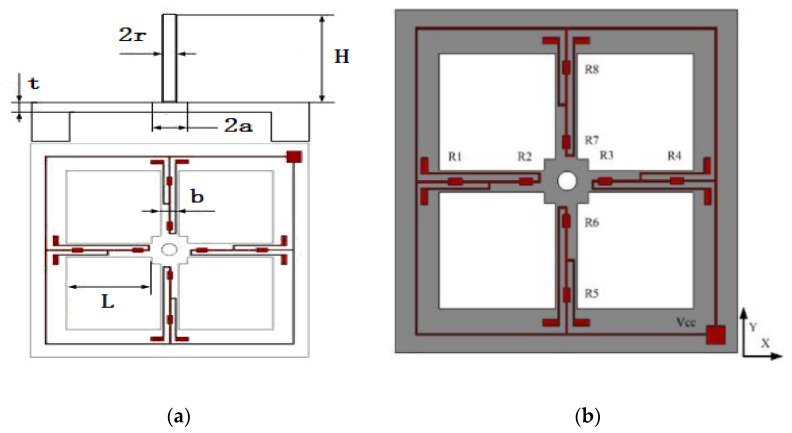
Structure and parameters of the microstructure. (**a**) Device structure (**b**) Distribution of piezoresistors.

The MEMS vector hydrophone which is fabricated by the team of Wendong Zhang is composed of a cilia bionic hydrophone and an acoustic pressure hydrophone. The cilia bionic hydrophone mimics the structure of neuromast in the fish lateral line. Its working principle is that sound waves act on the bionic cilia and make the bionic cilia offset to cause the deformation of the silicon cantilever beam structure. Then the resistance change in the beam is output by the Wheatstone bridge. It is a two-dimensional vector hydrophone whose outputs are the two components of the vibration velocity in the plane: V_X_ and V_Y_. The main design parameters of the microstructure are given in Figure 1 and Table 1.

**Figure 2:**
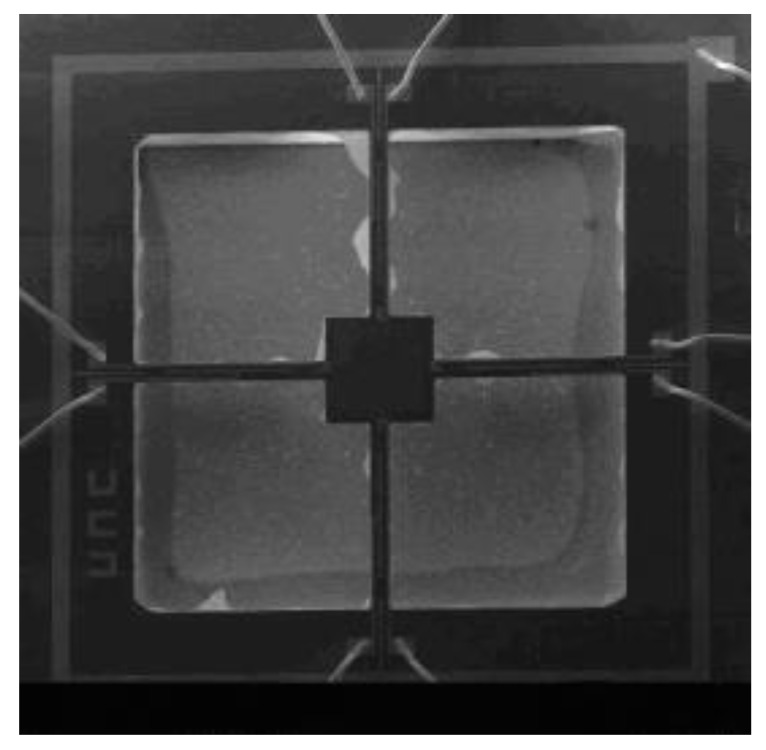
The microscope image of micro electromechanical system (MEMS) chip.

In Figure 1b, the gray part is the cross beam structure after etching; R1, R2, … R8 are the piezoristors on each beam, the red part is metal; the metal part marked as VCC is a pad which should be connect to the power. The specific manufacture process of MEMS chip refers to Xue’s research [13]. The microscope image of MEMS chip is shown in Figure 2.

**Figure 3:**
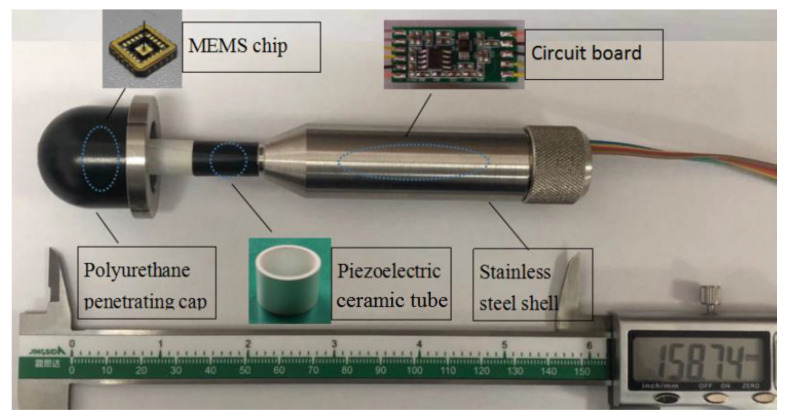
The picture of MEMS vector hydrophone and its composition.

The acoustic pressure hydrophone is composed of piezoelectric ceramic tube whose model is YT-5. When the external sound pressure acts on the piezoelectric ceramic tube, the inner and outer surfaces of the ceramic tube generate charges with opposite sign. The picture of MEMS vector hydrophone and its composition are shown in Figure 3. The MEMS vector hydrophone is composed of MEMS chip, piezoelectric ceramic tube, circuit board, etc. The circuit board uses the instrument amplifier INA163 to amplify the weak signal, which is placed inside the stainless steel shell.

### 2.2. Characteristics of the MEMS Vector Hydrophone

**Figure 4:**
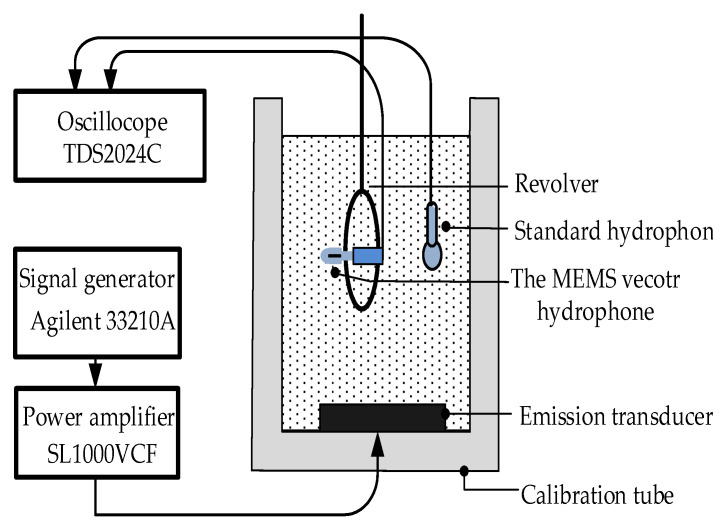
The actual test diagram of calibration equipment.

The characteristics of the MEMS vector hydrophone have been tested in a standing wave tube calibration equipment which is shown in Figure 4. In the equipment sine wave generated by signal generator is sent to emission transducer after amplification. The standard hydrophone is plunged in water and the tested hydrophone is fixed on the revolver.

The sensitivity can be obtained by standard comparison method through the following Equation [2]:

(1) $$M_{x}=\frac{U_{x}\sin{\left(kd\right)}_{}}{P_{0}\cos(kd)}$$

Wherein, M*_x_* is the sensitivity. *U_x_*, *P*_0_ is the output peak voltage of the tested hydrophone and standard hydrophone, respectively, *k* is the wave number, *d* is distances from the liquid level to hydrophones.

In the calibration tube, the acoustic pressure value can be expressed as P∝sin(kd), and 8-shaped directivity can be obtained through the following equation.

(2) $$D=20\log\frac{V_{\theta }}{V_{\max}}$$

where *V_θ_* is the output voltage at angle *θ*, and *V*_max_ is the maximum output voltage at all angles.

**Figure 5:**
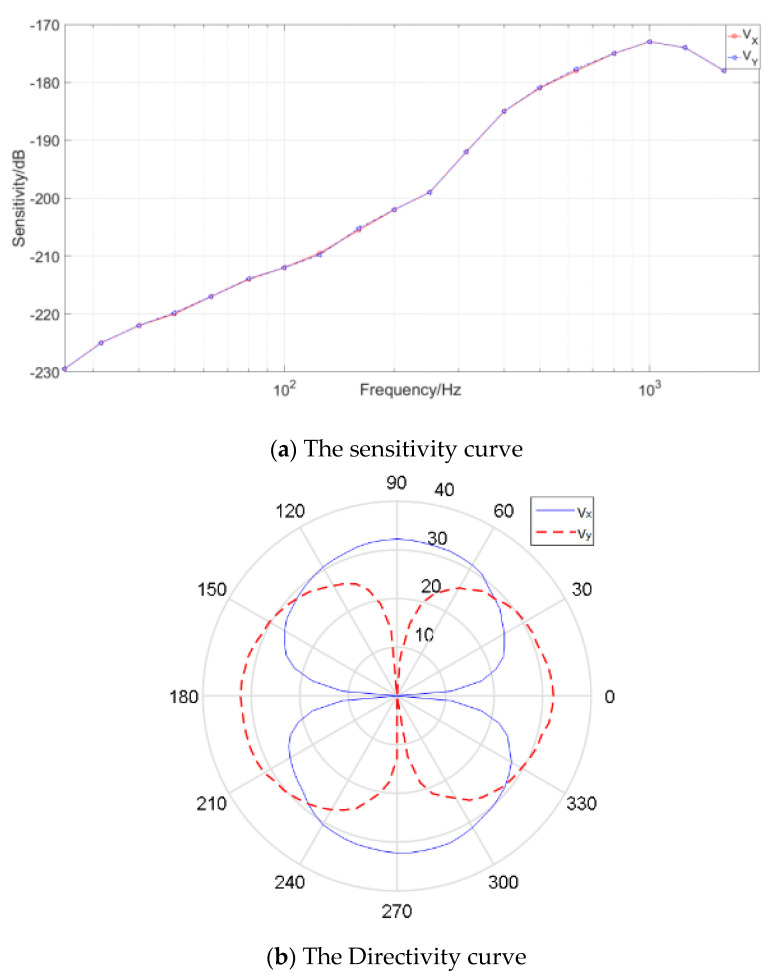
The test results of the MEMS vector hydrophone.

The test results are shown in Figure 5. As shown in Figure 5a, the hydrophone has good low frequency performance, its working frequency band is 20~1000 Hz, and has a high sensitivity: −181 dB at 500 Hz. The 100 Hz concave point depth of 8-shaped directivity is beyond 30 dB of acoustic vibration velocity as shown in Figure 5b.

### 2.3. Acoustic Energy Flux Detection Method

**Figure 6:**
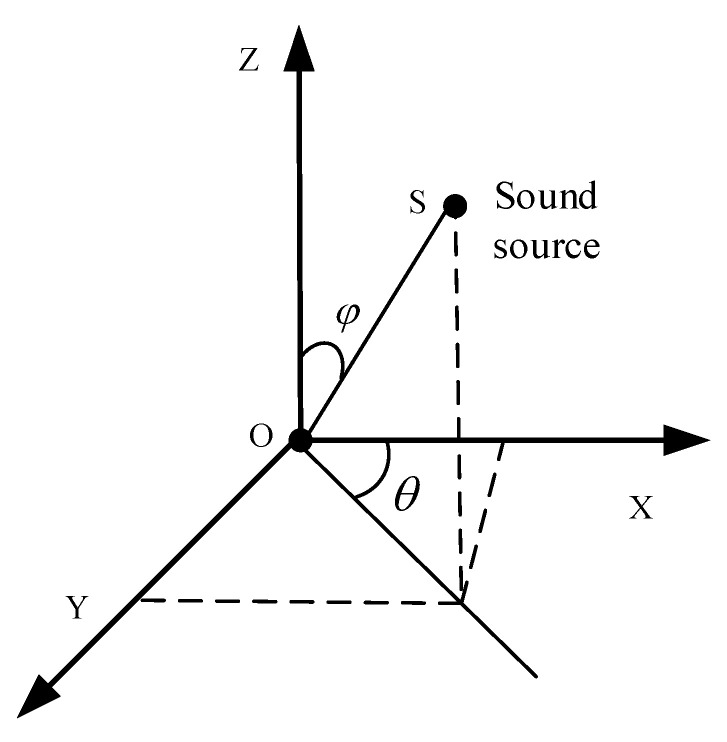
The scheme of direction of arrival (DOA) estimation using a vector hydrophone.

The scheme of DOA estimation using a vector hydrophone is shown in Figure 6. In the figure, S is the sound source, φ is the pitch angle of the incident direction of the sound source, *θ* is the horizontal angle. The vector hydrophone is located at the origin. Since MEMS vector hydrophone is a two-dimensional vector hydrophone, the true value of DOA estimation is *θ*.

The MEMS vector hydrophone obtains three acoustic quantities: *P*-acoustic pressure value, *V_x_* and *V_y_*-two orthogonal components of acoustic vibration velocity vector **V**. When the sensitivity coefficient is ignored, the expression of the output signal of the vector hydrophone is shown in Equation (3).

(3) $$\{\begin{array}{c} P=P\\ V_{x}=|V|\cos\theta \\ V_{y}=|V|\sin\theta \end{array}$$

Within unit time, the acoustic energy passing through unit area perpendicular to the direction of energy transmission is called the acoustic energy flux density, which is expressed as ω [14]. It is the product of acoustic pressure and vibration velocity, and its direction is the same as the vibration velocity. It can be seen from the definition that the value of acoustic energy can be obtained by time integral, that is, the acoustic energy flux ***I***, and its expression is shown in Equation (4).

(4) $$I=\int_{0}^{T}\omega (t)dt$$

wherein, ω(t)=P(t)V(t), ***T*** is observation time.

At time t, the acoustic energy flux density ω(t) can be regarded as the resultant vector of the acoustic energy flux density in all directions where sound source exist, and Equation (4) can be transformed into the form of Equation (5).

(5) $$I=\int_{0}^{T}\int_{0}^{2\pi }\omega (t,\theta )d\theta dt$$

wherein, ω(t,θ) is the acoustic energy flux density at time t in θ.

Equation (5) can be obtained by replacing the integral sequence and coordinate transformation of Equation (6).

(6) $$I=\int_{0}^{2\pi }\int_{0}^{T}\omega (t,\theta )dtd\theta =\int_{0}^{2\pi }\int_{0}^{T}\omega (\rho ,\theta )dtd\theta$$

In Equation (6), the polar coordinate form ω(ρ,θ) of acoustic energy flux density is used after the second equal sign, which represents the instantaneous acoustic energy flux density with module value of *ρ* and direction of *θ*. As can be seen from Equation (6), the acoustic energy flux density in each direction is integrated in the time domain to obtain the energy of each direction, and then the acoustic energy flux is obtained by adding the energy in all directions.

Equation (6) is transformed into the frequency domain to obtain Equation (7).

(7) $$I=\frac{1}{2\pi }\int_{0}^{2\pi }\int_{0}^{F}\omega (\rho ,\theta )dfd\theta$$

In Equation (7), ω(ρ,θ) represents the acoustic energy flux density in frequency domain with module value of *ρ* and direction of *θ*. Equation (7) decomposes the acoustic energy flux of each direction into the frequency domain. As can be seen from Equation (7), the acoustic energy flux density in each direction is integrated in the frequency domain to obtain the energy of each direction, and then the acoustic energy flux is obtained by adding the energy in all directions.

The vector hydrophone cannot directly output the acoustic energy flux density, but it can use its three outputs as parameters to get the acoustic energy flux density. The mode of the acoustic energy flux density can be obtained by Equation (8) [15].

(8) $$\rho (t)=\sqrt{I^{2}{}_{x}(t)+I^{2}{}_{y}(t)}$$

wherein, $I_{x}(t)=P(t)V_{x}(t)$, $I_{y}(t)=P(t)V_{y}(t)$.

The direction of the acoustic energy flux density can be obtained by Equation (9), which is the maximum likelihood estimation of the target direction [16]

(9) $$\theta (t)=\arctan[I_{y}(t)/I_{x}(t)]$$

Equations (8) and (9) are transformed into the frequency domain to obtain Equations (10) and (11).

(10) $$\rho (f)=\sqrt{I^{2}{}_{x}(f)+I^{2}{}_{y}(f)}$$

(11) $$\theta (f)=\arctan[I_{y}(f)/I_{x}(f)]$$

In the ocean channel, the acoustic Ohm’s law is approximately satisfied, and the acoustic pressure and the vibration velocity are in phase. According to the basic characteristics of the Fourier transform, the energy of the two inputs in the same phase is concentrated in the real part of the cross spectrum. Hence, in Equations (10) and (11), $I_{x}(f)=re(P(f)V_{x}{}^{*}(f))$, $I_{y}(f)=re(P(f)V_{y}{}^{*}(f))$.

Therefore, when the bandwidth of the sound source is known, a single MEMS vector hydrophone can be used to estimate the direction of target by acoustic energy flux detection by Equation (11).

## 3. The Weighted Histogram Algorithm

### 3.1. The Flow and Analysis of the Algorithm

**Figure 7:**
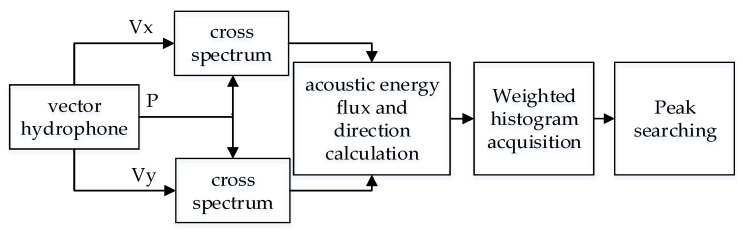
The signal processing flow of weighted histogram algorithm.

When the bandwidth of sound source is unknown, the direction resolution of multi-target can be realized in frequency domain based on acoustic energy flux detection. According to Section 2, the mode of acoustic energy flux density and directions at each frequency can be obtained. By integrating the acoustic energy flux densities in different directions, the energy in each direction, that is, the weighted histogram, can be obtained. It is generally believed that the direction of peak energy is the target direction. The signal processing flow of the weighted histogram algorithm is shown in Figure 7 [17].

In the algorithm, the output of the vector hydrophone is Fourier transformed to obtain the cross spectrum of sound pressure and vibration velocity. After calculating the acoustic energy flux and corresponding direction on each frequency component, the weighted histogram is obtained by statistical with equal interval angle. When the statistical interval is Δθ, the statistic is performed with 0° as the starting point. The statistical intervals are: 0~Δθ, Δθ~2Δθ, … (N−1)Δθ~NΔθ. A total of 180/Δθ times are counted, and the statistical method is shown in Equation (12).

(12) $$\left\{ \begin{array}{l} R(0\sim \Delta \theta )=\sum_{1}^{m_{1}}R_{r}(f)\\ R(\Delta \theta \sim 2\Delta \theta )=\sum_{1}^{m_{2}}R_{r}(f)\\ \ldots \ldots \\ R[(N-1)\Delta \theta \sim N\Delta \theta ]=\sum_{1}^{m_{N}}R_{r}(f) \end{array}\right.$$

wherein, *m*_1_, *m*_2_, … *m_N_* are the number of frequency points falling into the corresponding statistical interval, $R_{r}(f)$ is the module value of the acoustic energy flux within the range of the statistical interval. From Equation (12), the distribution of the energy in each statistical interval can be obtained, that is, the weighted histogram of the acoustic energy flux. Finally, the peak search is performed in the weighted histogram, and the angle of the statistical interval with the largest energy is considered as the result of the DOA estimation.

In this paper, the environmental noise is regarded as an isotropic Gaussian white noise. In such a noise field, the acoustic pressure output of the vector hydrophone is white Gaussian noise P~N(0,*σ*^2^), and the vector output follows the Gaussian distribution: *V_x_*~N(0,*σ*^2^/2), *V_y_*~N(0,*σ*^2^/2), which are independent of each other. When Equations (8) and (9) are applied to calculate the acoustic energy flux and its direction, the components of vector acoustic energy flux density *I_x_* and *I_y_* must be obtained first, which are obtained by the product of the acoustic pressure and the corresponding vibration velocity component. Since the two product factors of the acoustic energy flux density are both Gaussian distributions, their products are also Gaussian distributions [18], and *I_x_*~N(0,*σ*^2^/3), *I_y_*~N(0,*σ*^2^/3).

According to the definition of the Rayleigh distribution, when the two components of a random two-dimensional vector are independent, with a mean value of 0, and have the same normal distribution of variance, the modulus of this vector is a Rayleigh distribution. It can be seen from Equation (10) that the mode of acoustic energy flux is Rayleigh distribution [19], and its probability density function is:

(13) $$f(t)=\frac{t}{\sigma^{2}/3}e^{-\frac{t^{2}}{2\sigma^{2}/3}}$$

Its mathematical expectation is shown in Equation (14).

(14) $$\begin{array}{ll} E(t) & =\int_{0}^{\infty }tf(t)dt\\ & =\int_{0}^{\infty }\frac{t^{2}}{\sigma^{2}/3}e^{-\frac{t^{2}}{2\sigma^{2}/3}}dt\\ & =\sqrt{\pi /6}\sigma \end{array}$$

Its variance is shown in Equation (15).

(15) $$\begin{array}{ll} \mathrm{var}(t) & =\int_{0}^{+\infty }t^{2}\frac{t}{\sigma^{2}/3}e^{-\frac{t^{2}}{2\sigma^{2}/3}}dt-u^{2}(t)\\ & =\frac{12-\pi }{6}\sigma^{2} \end{array}$$

Therefore, the expectation and variance of the acoustic energy flux model are both positively correlated with the noise energy. The greater the noise, the greater the expectation and variance of the corresponding acoustic energy flux.

Since the two factors of division are both normal distributions with an expectation of 0, the result of *I_y_*/*I_x_* following the Cauchy distribution [20]. The arc tangent function, that is, the result of Equation (11) is uniformly distributed, and $\theta (t)\sim U(-\frac{\pi }{2},+\frac{\pi }{2})$ according to the nature of the Cauchy distribution [21]. Due to the transitivity of the Gaussian distribution, in the frequency domain, the above conclusion is still valid. In other words, for noise, the direction of each frequency component is uniformly distributed, and the acoustic energy flux follows the Rayleigh distribution.

When there is a target with a certain strength in the noise field, the acoustic energy flux is the superposition of noise and the acoustic energy flux radiated by the target. At this moment, the direction calculation result obtained by the average sound intensity device is or close to Gaussian distribution [22], whose mathematical expectation is true direction. Therefore, when the target radiates the broadband signal, the result of DOA estimation of each frequency component in the target bandwidth is also or close to Gaussian distribution. Each frequency component outside the target bandwidth contains only noise, and the result of DOA estimation is still uniformly distributed. It can be seen that, on the weighted histogram, the energy will accumulate in and near the real target direction, forming a bell-shaped curve, and the peak value is ideally the real target direction. However, because there is uniformly distributed noise acoustic energy flux in each direction, when the noise power is great, there is a certain probability that the sound source energy and the noise are superimposed to form a false peak near the true direction. In this case, the direction of the energy peak will deviate from the true direction of the target.

### 3.2. Physical Model and Simulation

**Figure 8:**
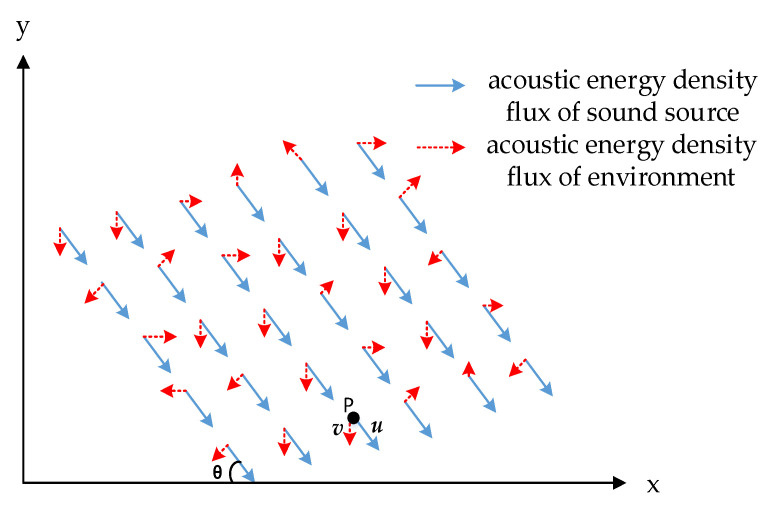
The instantaneous physical model of the sound field.

When the sound source is in the far-field, the acoustic energy flux density at a point in the space can be regarded as the sum of the acoustic energy flux density of the sound source and that of the noise. The instantaneous physical model of the sound field near the vector hydrophone is shown in Figure 8.

In Figure 8, the angle between the direction of the sound source signal and the horizontal direction is *θ*. The blue arrows are the sound energy flow density vectors of the sound source, and the red ones are the sound energy flow density vectors of the noise at corresponding position. The vector hydrophone is placed at point P, and the sound energy flux density vector at P can defied to *w* and *w* = *u* + *v*, where *u* is the sound energy flux density of the sound source, and *v* is that of the noise. The direction of the sound source signal is *θ*, which is the correct result of DOA estimation. In this paper, the environmental noise is assumed to be isotropic white Gaussian noise, and the azimuth and value of the noise energy flux density are evenly distributed in different directions in different time and space. The direction of the resultant vector ***w*** will inevitably be affected by the noise and deviate from *θ*.

In order to verify the physical model and the derivation of Section 3.1, the sound energy flow distribution at point P is simulated using Equation (11). The simulation conditions are: the SNR of point P is 0 dB, the noise is Gaussian noise, the sampling rate is 4 k, the sampling time is 1 s, a continuous spectrum broadband signal radiated by the target (10~400 Hz) with 30°, the number of simulation time is 10,000 and 100,000, respectively.

**Figure 9:**
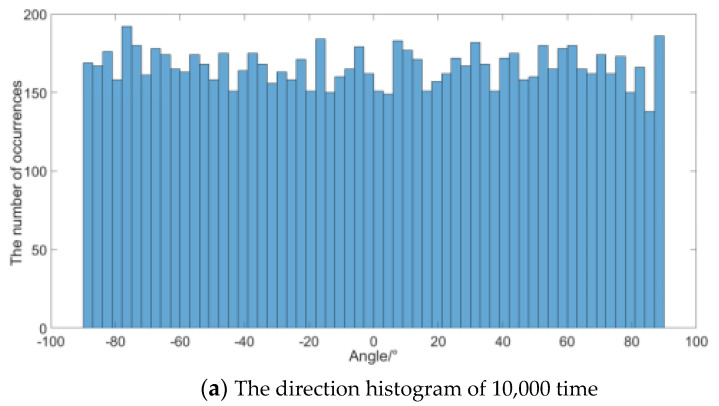

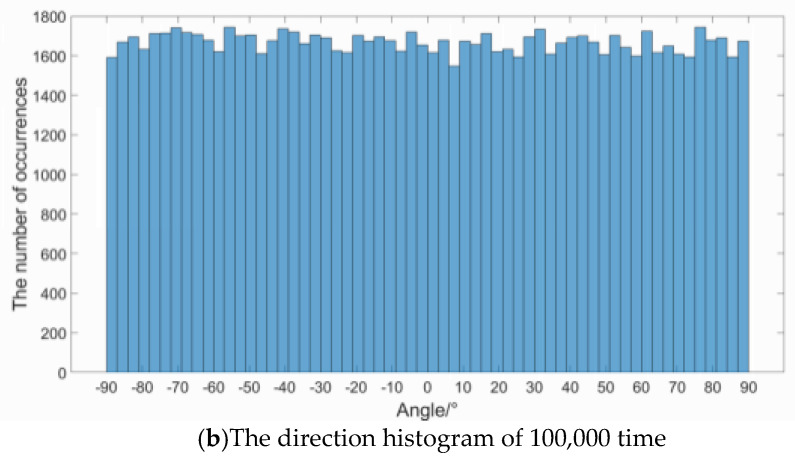
Simulated result at 800 Hz.

Histogram statistics is performed on the calculation results of the noise frequency (800 Hz here) in Figure 9. Figure 9a,b are the statistical results of 10,000 and 100,000 times, respectively. The simulation results show that the distribution of each angle is relatively uniform, which verifies that the sound energy flow density of the noise is uniformly distributed. The standard deviation of data in Figure 9a is 51.2, and that of Figure 9b is 48.3, and the sample size of the latter is 10 times larger. The results show that the statistical results of the distribution are more accurate with the simulation times increase and the directional distribution of noise close to uniform.

**Figure 10:**
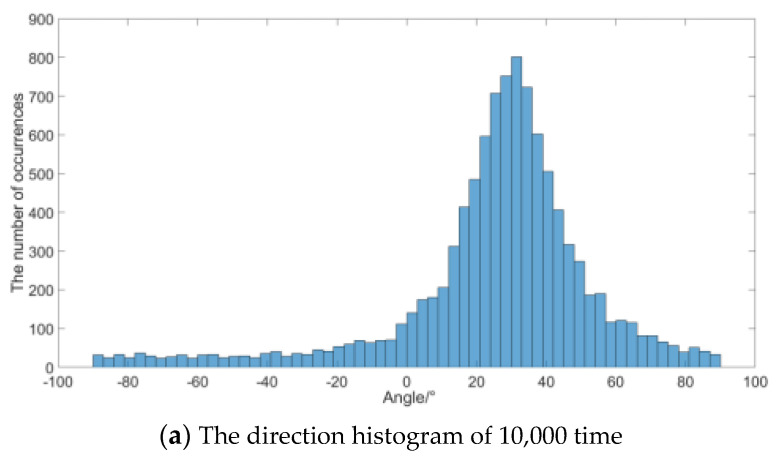

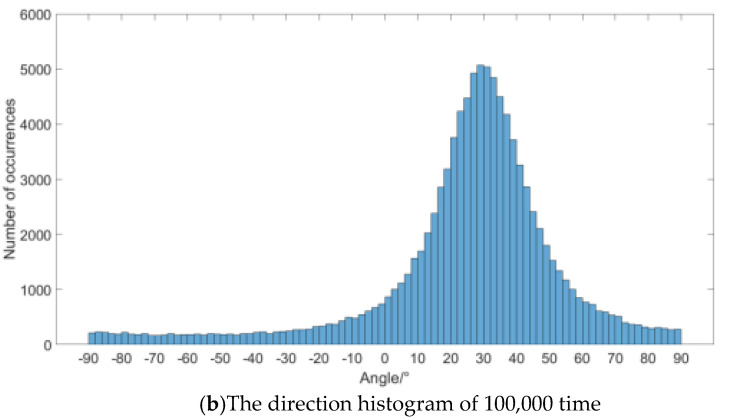
Simulated result at 300 Hz.

Histogram statistics is performed on the calculation results of the signal frequency (300 Hz here) in Figure 10. Figure 10a,b are the statistical results of 10,000 and 100,000 times. It can be seen from the figure that the mean value of the statistical distribution is 30°, and the bell curve of normal distribution is very obvious near 30°. Figure 10b is smoother obviously, which shows that the distribution results are more accurate when the simulation times increase. The result verifies that the direction of sound energy flow density under the influence of noise is close to normal distribution. When the number of statistics is enough, the maximum number of statistics can be the direction of the sound source, but the weighted histogram algorithm needs to accumulate the sound energy value of each direction including the noise. So when the integration time is limited, the peak value may deviate from the true value, for example the result of a single statistic in Figure 10b may be any direction.

**Figure 11:**
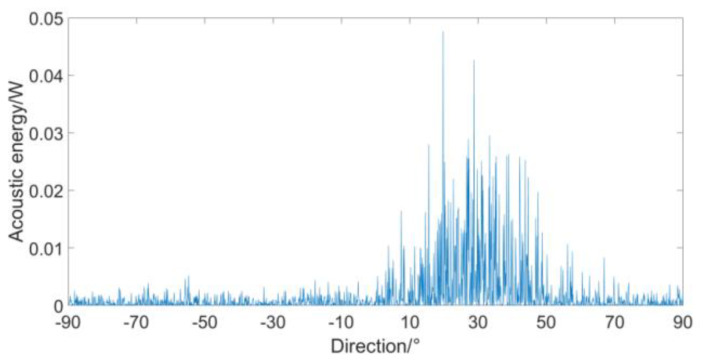
Simulated result of acoustic energy flux distribution.

In this paper, the principle of the weighted histogram algorithm is used to simulate the spatial distribution of the acoustic energy flux as follows. The simulation conditions are: the SNR is 0 dB, the noise is Gaussian noise unrelated with the target, the sampling rate is 4 k, the sampling time is 1 s, a continuous spectrum broadband signal radiated by the target (10~400 Hz) with 30°, and the statistical interval is 0.1°. The simulated weighted histogram is shown in Figure 11.

In Figure 11, the peak is not the target direction. Near the true direction, an acoustic energy flux distribution with an envelope similar to a bell shape is formed, but the peak is not sharp, and great acoustic energy flux appears in multiple directions. Therefore, in the original weighted histogram algorithm, the peak searching is defective to determine the target direction.

## 4. The Improved Weighted Histogram Algorithm

**Figure 12:**
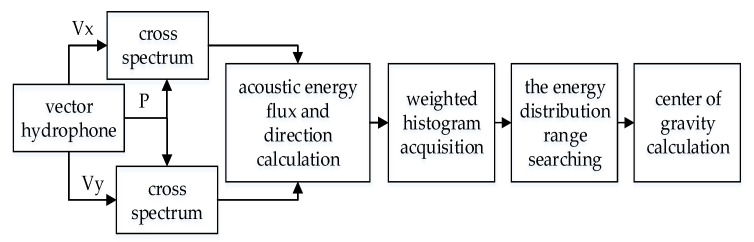
The signal processing flow of the improved algorithm.

It can be seen from Section 3 that the target direction is the mathematical expectation value of its acoustic energy flux distribution. This paper proposes an improved weighted histogram algorithm based on this principle. In this algorithm, the weighted histogram is obtained in the same way. Then the peak searching in the original algorithm is replaced by the energy distribution range searching and center of gravity calculation. The signal processing flow is shown in Figure 12.

**Figure 13:**
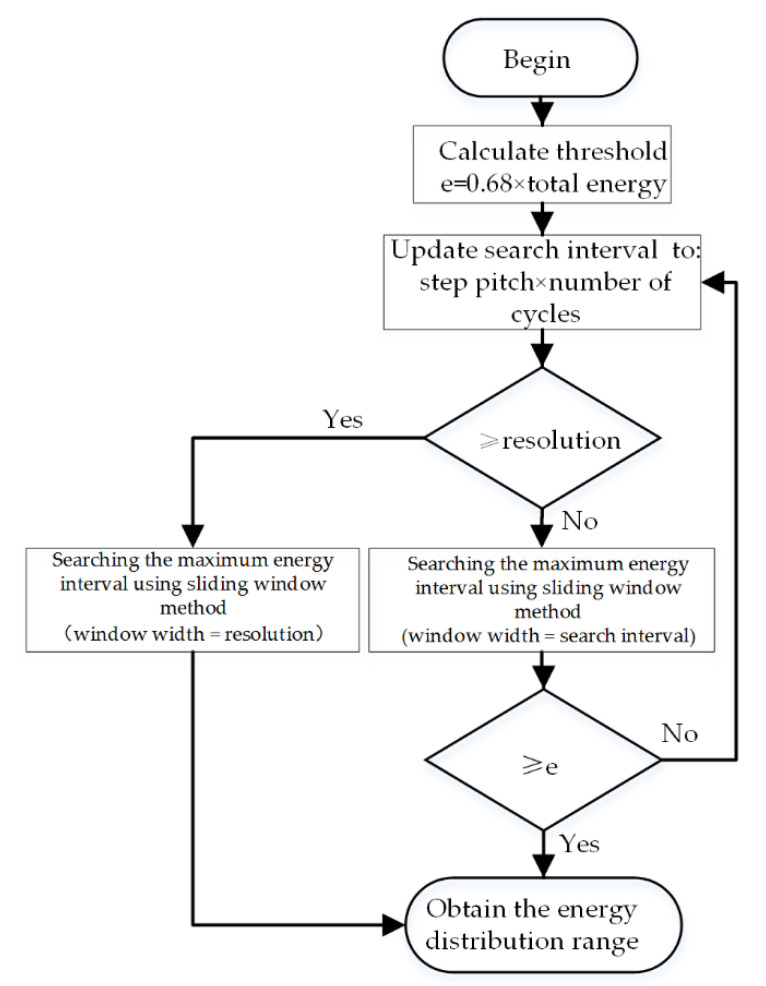
The search process of the energy distribution range.

The search for the distribution range is realized by a variable sliding window. In this way, the minimum distribution interval where the total energy reaches a threshold is found in the weighted histogram, and this interval is regarded as the range of the sound source energy distribution. In this paper, 68.2% of the total energy value is taken as the threshold, which is the probability that the value in the normal distribution falls within the range of (μ±σ). The obtained interval can represent the range of most target energy. The resolution of the algorithm refers to the minimum angle between two targets which the algorithm can distinguish. Only one result of DOA estimation can be obtained in the minimum distribution interval, thus it is necessary to set the interval not to be greater than the algorithm resolution required by the usage conditions. The search process of the sound source energy flux distribution range is shown in Figure 13.

As shown in Figure 13, when the search range is determined by the product of the step pitch and the number of cycles, and the maximum value is the resolution required by the algorithm. The variable sliding window is adopted to search. When the search range is φ, the search process is: the sliding step pitch is Δθ (the same as the statistical interval of the weighted histogram), the starting point of the statistical interval starts from 0°, and Δθ is increased each time. The end point of the interval is always φ greater than the starting point, so that 180/Δθ statistical intervals are obtained. The acoustic energy flux in each interval is accumulated to find the maximum value. When the result is greater than or equal to the threshold, the statistical interval is considered as the minimum distribution interval.

After the target acoustic energy flux distribution range is obtained, the target direction is calculated by the energy center of gravity method in this range, and this direction is the result of DOA estimation. The energy center of gravity method is based on the energy center of gravity characteristic of the power spectrum of the symmetric window function, and the method used is shown in Equation (16).

(16) $$\psi =\frac{\sum K_{i}\theta_{i}}{\sum K_{i}}$$

wherein, *K_i_* is the mode of acoustic energy flux radiated by the target when the direction is *θ_i,_ ψ* is the result of DOA estimation.

When there is noise, Equation (16) can be converted to Equation (17).

(17) $$\psi =\frac{\sum (I_{i}+J_{i})\theta_{i}}{\sum \left(I_{i}+J_{i}\right)}=\frac{\sum I_{i}\theta_{i}}{\sum I_{i}+\sum J_{i}}+\frac{\sum J_{i}\theta_{i}}{\sum I_{i}+\sum J_{i}}$$

wherein, *I_i_* is the mode of acoustic energy flux radiated by the target in the *θ_i_* direction, and *J_i_* is the mode of acoustic energy flux of the noise in the *θ_i_* direction. It can be seen from the algorithm flow that energy flux distribution range has been limited to the interval where the target acoustic energy flux is concentrated. When $\sum I_{i}>>\sum J_{i}$ is met within this interval, Equation (17) can be converted to:

(18) $$\psi =\frac{\sum I_{i}\theta_{i}}{\sum I_{i}}+\frac{\sum J_{i}\theta_{i}}{\sum I_{i}}$$

The numerator in the first term on the right side of Equation (18) can be decomposed into the product of acoustic energy flux mode value in each frequency component and its direction of the target. The denominator is the sum of the acoustic energy flux mode value in each frequency component. When the target energy is basically flat within the bandwidth, Equation (18) can be converted to:

(19) $$\psi =\frac{I\sum \theta_{i}}{NI}+\frac{\sum J_{i}\theta_{i}}{\sum I_{i}}=\bar{\theta }+\Delta \theta$$

Wherein, *I* is the acoustic energy flux mode value of each frequency component, and *N* is the number of frequency points. According to the analysis in Section 3, *θ_i_* is Gaussian distribution, so the average value $\bar{\theta }$ obtained is the maximum likelihood estimation of the target direction. $\Delta \theta =\frac{\sum J_{i}\theta_{i}}{\sum I_{i}}$ is the error value. The error of the algorithm is negatively related to the SNR in the selected area. The higher the SNR, the smaller the error. When the error is negligible, the result of Equation (19) can be considered as the maximum likelihood estimation.

## 5. Experiment and Result Analysis

### 5.1. Simulation Test

In terms of the improved algorithm, the resolution of the algorithm is closely related to the performance of the algorithm. The excessively large resolution will affect the multi-target resolution ability because the algorithm can only distinguish one sound source in a searching interval. The excessively small value will cause the small energy in the obtained interval, that is, reducing the number of samples for maximum likelihood estimation, thereby reducing the estimation accuracy.

**Figure 14:**
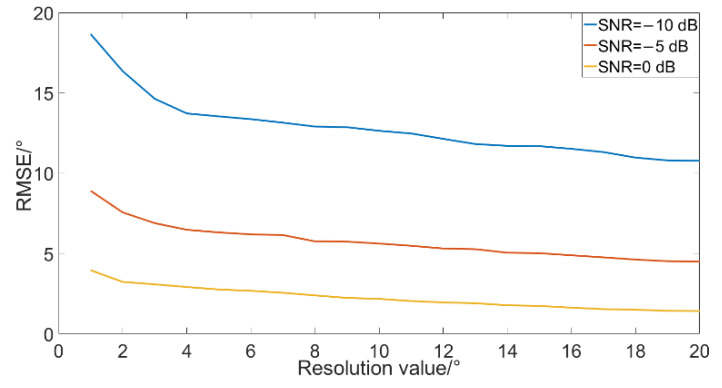
Simulation results in different resolution.

The simulation of the results of DOA estimation when the resolution changes under different SNR is performed as follows. The simulation conditions are: the SNR is −10 dB, −5 dB and 0 dB, the noise is Gaussian white noise, the sampling rate is 4 k, the sampling time is 1 s, the continuous spectrum broadband signal radiated by target (10~400 Hz) with 30°, the statistical interval is 0.1°, and the resolution is 1°~20°. At each resolution, the number of simulations is 1000, and the simulation results are shown in Figure 14.

It can be seen from Figure 14 that the resolution has a great influence on the algorithm when the SNR is −10 dB. Resolution range increases the accuracy of the algorithm under all SNR, but the influence gradually becomes less when the SNR is −5 dB and −10 dB. When the resolution is greater than 10°, the result of DOA estimation gradually stabilizes. In order to ensure the algorithm still has a certain ability of DOA estimation and multi-target distinguishing, the resolution of the improved algorithm can be 10°.

**Figure 15:**
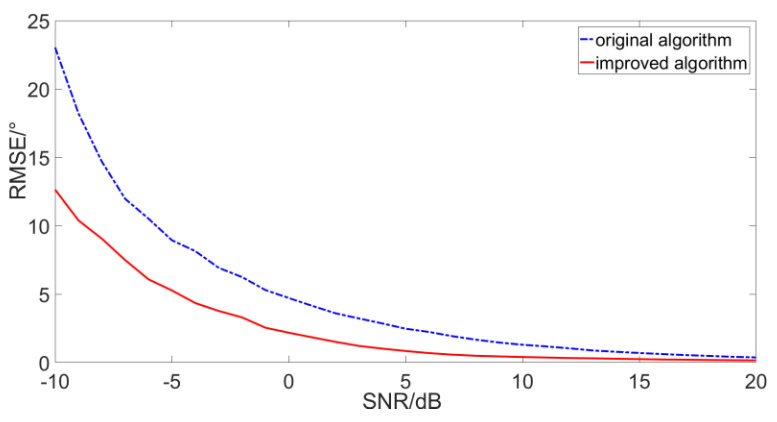
Simulation results in different signal to noise ratio (SNR).

In order to verify the effectiveness of the improved algorithm, the performance of DOA estimation for the improved algorithm and the original algorithm are simulated and compared under different SNR. The simulation conditions are: the SNR is −10 dB~20 dB, the noise is Gaussian white noise, the sampling rate is 4 k, the sampling time is 1 s, the continuous spectrum broadband signal radiated by target (10~400 Hz) with 30°, the statistical interval is 0.1°, and the resolution is 10°. At each SNR, the number of simulations is 1000, and the simulation results are shown in Figure 15.

As shown, when the SNR is −10 dB to 20 dB, the results of DOA estimation of the two algorithms improve with the increase in the SNR, and the results of the improved algorithm are better than the original algorithm in each SNR. The RMSR is reduced by 47.8% on average. Especially, when the SNR is 0 dB, the experimental results of the original algorithm and the improved algorithm are 4.71°and 2.17°, respectively, and the performance of the improved algorithm is improved by 2.54°.

**Figure 16:**
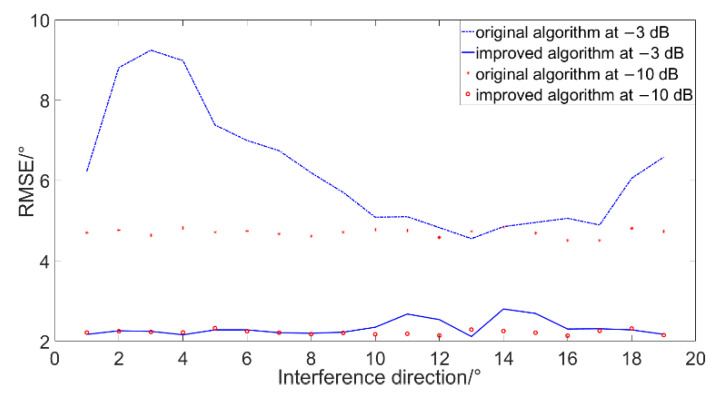
Simulation results in different interference direction and SNR.

In order to verify the performance of the algorithm in the environment with interference, the improved algorithm is compared and simulated with the original algorithm when there is a target and an interference signal. The simulation conditions are: the power ration of target to noise is 0 dB, the noise is Gaussian noise, the sampling rate is 4 k, the sampling time is 1 s, the continuous spectrum broadband signal radiated by target (10~400 Hz) with 30°, and the statistical interval is 0.1° and the resolution is 10°. The interference signal is a continuous spectrum broadband signal (600~1000 Hz) different from the target frequency band. The power ratio of interference to noise is −10 dB and −3 dB, respectively. The simulations are performed when the interference direction is changed from −90° to 90°. The simulations are performed for 1000 times, and the simulation results are shown in Figure 16.

The simulation results show that when the interference signal power is low (SNR is −10 dB), it has less of an effect on the results of DOA estimation of the two algorithms. When the interference signal power is high (SNR is −3 dB), the RMSE of the improved algorithm only increases when the interference signal is near the target, but the RMSE of the original algorithm gradually increases as the interference signal is far away from the target. The results of DOA estimation of the improved algorithm are better than the original algorithm in the above cases. When the power ratio of interference to noise is −10 dB and −3 dB, the RMSE is reduced by 54.5% and 62.6% on average, respectively.

### 5.2. Experiment under Lake Conditions

**Figure 17:**
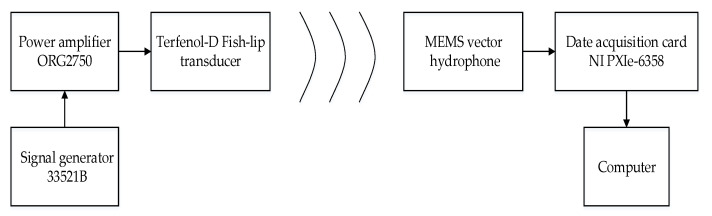
The testing system scheme.

In order to test the algorithm in a real environment, the testing system scheme is built as shown in the Figure 17. The sound source consists of a fish lip transducer connected with a signal generator and power amplifier. A MEMS vector hydrophone is connected with the data acquisition card, which converts the analog signal from the hydrophone into digital signal and sends data to the computer.

**Figure 18:**
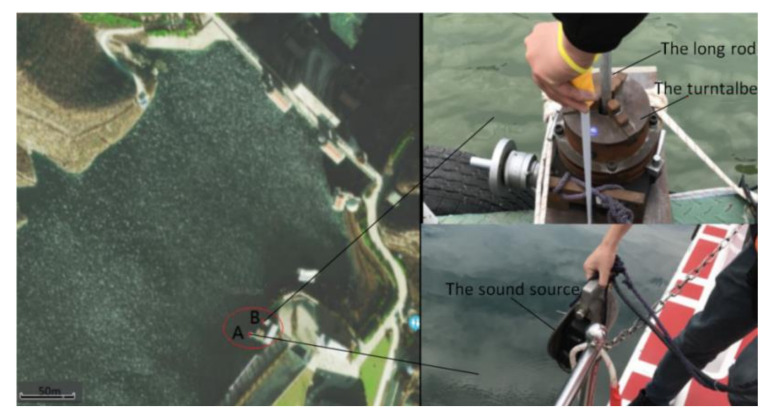
The experimental environment and equipment.

The experiment is carried out in a lake. The water surface of the selected lake is relatively open, with an average depth of more than 30 m. The experimental environment and equipment are shown in Figure 18.

**Figure 19:**
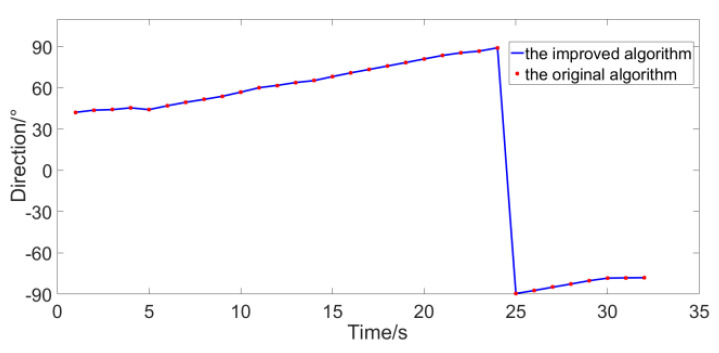
The experimental results under lake water conditions.

In Figure 18, the experimental platform composed of floating bridges is in the red circle. The sound source is lifted into the water from point A. A single MEMS vector hydrophone is fixed at the end of a long road and connected with the center of a turntable which can be rotated, and it is put into the water from point B. They are all placed 5 m underwater and the distance between them is 15 m. In the experiment, the continuous spectrum broadband signal (0~400 Hz) is sent out by sound source and the vector hydrophone is rotated by 60°. The signal acquisition card is used to sample three channels of the vector hydrophone. The sampling rate is 4 K. The original algorithm and the improved algorithm are used for calculations, respectively. The statistical interval is 0.1° and the resolution is 10°. The experimental results are shown in Figure 19.

**Figure 20:**
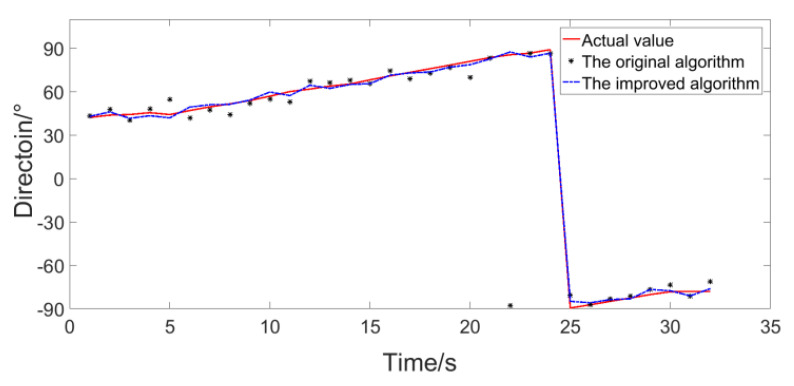
The experimental results after reducing the SNR.

The distance between the sound source and the vector hydrophone is relatively close, and the SNR reaches 41 dB. Under such a high SNR, we use the method shown in Figure 19 for simulation, and the RMSE the original algorithm and the improved algorithm are both 0.1° which has reached the statistical interval. Therefore, the variation range of the trajectory angle obtained by the two algorithms is consistent with the actual situation and very close, which is consistent with the conclusion in the previous section. In order to verify the performance of the algorithm in low SNR, the noise collected in the experimental site is amplified and added to the collected signal (the original signal is regarded as pure signal) to reduce the SNR to 0 dB. The same method is used for calculation, and the results in Figure 19 are compared as the true values, and the results are shown in Figure 20.

In Figure 20, the red solid line is the result of the improved algorithm when no noise is added, and it is taken as the true value here. As shown, when the SNR is reduced, the trajectory obtained by the improved algorithm is closer to the true value. The RMSE of the original algorithm and the improved algorithm are 4.78°and 2.21°, respectively. Therefore, the improved algorithm has better performance under the experiment conditions and the results are consistent with the simulation results in Figure 15.

## 6. Conclusions

The acoustic energy flux detection principle and the weighted histogram for DOA estimation using a single MEMS vector hydrophone are studied in this paper. The data processing steps of weighted histogram algorithm are analyzed in detail. Combined with mathematical reasoning and simulation, it is found that the “peak searching” is unreasonable. In terms of problems existing in the original algorithm, an improved weighted histogram is proposed. The energy center of gravity in the range is considered as the result of DOA estimation in improved algorithm and its performance is verified via simulation and experiment. The simulation results show that the RMSE of the improved algorithm is 47.8% lower than that of the original algorithm when the SNR is −10 dB~20 dB. The improved algorithm has better performance than the original one, and experiments in real environment also verify this conclusion.

## Figures and Tables

**Table 1:** The main parameters of vector hydrophone unit.

Parameters	Symbol	Value
Beam length	L	1000 µm
Beam width	b	120 µm
Beam thickness	t	40 µm
Center connector side length	2a	600 µm
Center connector thickness	t	40 µm
The cilia height	H	4000 µm
The cilia radius	r	150 µm
